# Early-stage visual perception impairment in schizophrenia, bottom-up and back again

**DOI:** 10.1038/s41537-022-00237-9

**Published:** 2022-03-21

**Authors:** Petr Adámek, Veronika Langová, Jiří Horáček

**Affiliations:** 1 Third Faculty of Medicine, Charles University, Prague, Czech Republic; 2 Present Address: Center for Advanced Studies of Brain and Consciousness, National Institute of Mental Health, Klecany, Czech Republic

**Keywords:** Schizophrenia, Neural circuits

## Abstract

Visual perception is one of the basic tools for exploring the world. However, in schizophrenia, this modality is disrupted. So far, there has been no clear answer as to whether the disruption occurs primarily within the brain or in the precortical areas of visual perception (the retina, visual pathways, and lateral geniculate nucleus [LGN]). A web-based comprehensive search of peer-reviewed journals was conducted based on various keyword combinations including schizophrenia, saliency, visual cognition, visual pathways, retina, and LGN. Articles were chosen with respect to topic relevance. Searched databases included Google Scholar, PubMed, and Web of Science. This review describes the precortical circuit and the key changes in biochemistry and pathophysiology that affect the creation and characteristics of the retinal signal as well as its subsequent modulation and processing in other parts of this circuit. Changes in the characteristics of the signal and the misinterpretation of visual stimuli associated with them may, as a result, contribute to the development of schizophrenic disease.

## Introduction

Schizophrenia affects a wide range of domains within information processing such as perception, thinking, attention, verbal fluency, working memory, executive functions, verbal memory, and learning^1^. These changes affect even the initial phase of information processing—perception, which is one of the fundamental tools humans use to learn about the world and adapt to its conditions. A disruption of the mechanisms involved in the processing of all perception modalities—olfactory^2^, somatosensoric^3^, auditory^4,5^, and visual percepts^4,6–8^—has been repeatedly demonstrated in schizophrenia.

Changes in visual perception in schizophrenia patients are apparent at the level of the oculomotor response to visual stimuli^9–11^. These disruptions also manifest as abnormalities in perceptional organization^12^, sensitivity to contrasts^7,13^, inaccurate perception of motion^14^, colors, brightness, distortion of shapes, and the disruption of perception of human figures and their emotional expressions^15^. These changes in visual perception are evident not only during acute schizophrenia episodes, but also in patients in remission^3,16^, and some may also be found in their relatives^17–19^. Congruently, longitudinal studies have shown the possibility to selectively predict the development of schizophrenia spectrum disorders in early adults based on measurements of the dysfunction rate in visual perception tasks performed by a high-risk child population^20,21^. Abnormalities of visual perception may, therefore, be considered as endophenotypes of schizophrenia^22,23^.

The incidence of disruption of visual perception in schizophrenia patients is high, ranging between 40 and 62%^24^, and has been described in the prodromal stage of the disorder^25^. Current views attribute impaired efficiency/functionality in visual perception processing in schizophrenia patients mainly to dopaminergic modulation of the incoming signal. This modulation is related to gain control and its subsequent integration into visual processing^26^.

One characteristic impairment is instability in the ability to process low spatial frequency (LSF) information from a visual scene. LSF information is rapidly extracted from a visual stimulus and provides general information about the shape and orientation of objects in a visual scene. Top-down prediction, which affects our visual attention and higher brain functions related to visual cognition, is then formed based on these LSF data^27–30^. In early-stage and untreated first-episode patients, hypersensitivity is often encountered, which eventually progresses to hyposensitivity, which also begins to extend to other frequencies of the visual scene^26,31,32^. The impairment of sensitivity to spatial frequencies is not limited to LSFs, however, and as the disease progresses, it begins to manifest in the middle and high spatial frequencies as well. LSFs probably occupy a specific place within visual information processing^28^.

One of the main consequences of the disruption of this process is a disorder of attention and the inability to integrate salient percepts into the stream of consciousness^33,34^. In schizophrenia, the occurrence of brain activation abnormalities (both hyper- and hypoactivations) in visual tasks has been described in temporal^35^, occipital^36,37^, parietal, and prefrontal^38,39^ areas, depending on specific experimental tasks. These tasks reflect both the disruption of the mechanisms of basal visual perception based on incorrect processing of visual stimuli (bottom-up)^7,13,37,40^ and the disruption of higher visual cognition based on the processing of visual stimuli influenced and orchestrated by previous experience (top-down/feedforward sweep)^6,41–44^. Errors in precortical areas of visual processing (the retina, optic nerve, thalamus) cause subsequent errors in higher cognitive processes. The decrease in the information flow in precortical visual pathways probably leads to a distorted condition where the brain evaluates and models a situation based on incomplete or incorrect input signals and is not able to properly modulate and integrate them into consciousness^5,45,46^. A low signal-to-noise ratio^47^ in particular results in an increased level of vagueness related to the nature of a percept/signal, leading to a disruption of the decision-making process^48^. This leads to compensation effects in the form of overlapping receptive fields of retinal cells, inhibition of visual information preprocessing caused by a higher number of errors, and excessive amplification of sensoric and noise signals^49^. This pathological process may also be facilitated by dopamine (DA)^50^, acetylcholine (ACh)^51^, and glutamate^52^ dysregulation modifying the electrophysiological response to stimuli.

Top-down modulation of visual perception is provided via several complementary mechanisms and, given the fact that the quality of visual perception affects higher cognitive functions, impairments in all of the modulatory mechanisms may give rise to various cognitive schizophrenia symptoms apparent in tasks challenging attention, working memory, or associative and executive functions. Attention and working memory are a basis for higher cognitive functions, such as associative and executive functions, and, vice versa, executive functions control attention and working memory in a feedback loop^53–55^.

Top-down control, as well as bottom-up processing of visual perception, may be disturbed by alterations in cortical and subcortical brain regions as documented in schizophrenia patients and also supported by animal models. In postmortem and brain imaging studies of schizophrenia patients, abnormalities including enlarged lateral ventricles and reductions in gray and white matter in subcortical and cortical regions were observed^56–58^. In rodent animal models, schizophrenia-like symptoms after lesions in the prefrontal cortex (PFC), ventral hippocampus (homological to the anterior hippocampus in humans), amygdala, or nucleus accumbens have been documented. Interestingly, these lesions resulted in altered connectivity or neurochemistry of the limbic circuit or modifications to the cytoarchitecture of the PFC^59–63^. Together, the aberrant early stages of visual processing represent the candidate mechanism for explaining the development of core schizophrenia symptoms.

The aims of this article are to: (1) summarize the current knowledge of visual perception impairment in schizophrenia patients on each level of the precortical visual signal processing pathway (the retina, optic nerve, LGN) and the effect of such impairments on visual perception, (2) compare pathophysiological alterations of the visual precortical pathway with cortical pathological changes documented in schizophrenia patients, and (3) propose a new context of schizophrenia symptoms stemming from the pathophysiology of the visual signal processing.

## Retina

Information about the external environment enters the visual system through the retina, where the early phases of input signal processing and transformation take place. The input signal is modulated by more than twenty types of ganglion cells responsible for converting visual information into an electrochemical signal, the characteristics of which correspond to various attributes of a visual percept^64^.

Abnormalities in retinal electrophysiological responses to stimulation by light^65^, morphological alteration of retinal structure^66^, and alterations of retinal metabolic processes^67^ may be found in schizophrenia patients. Although visual perception is one of the most intensively studied and well-understood fields of neuroscience, reports on the topic of retinal structure and function comprise only 2% of all studies of visual perception in schizophrenia^49^. At the same time, it is a malfunction of the retina that most often leads to lower sensitivity to contrast and to high spatial frequencies of an image stimulus^68–70^, distortion of color perception, reading issues^71,72^, and some types of visual distortion and hallucinations^73–75^ in schizophrenia patients. However, visual hallucinations, in particular, are a relatively rare symptom of schizophrenia and only occasionally appear alone; they are more often accompanied by hallucinations of other modalities^76^. This fact may suggest a common pathophysiological mechanism underlying hallucinations of visual and other perceptual domains. In comparison with only unimodal hallucinations, a combination of visual and auditory hallucinations is associated with an increased severity of gray matter volume (GMV) reduction. The reduction in GMV in first-episode patients with combined visual and auditory hallucinations is especially prominent in the occipital cortex and frontoparietal areas^77,78^. Interestingly, the severity of GMV reduction in certain areas is accompanied by increased functional connectivity and is related to the severity of both visual and auditory hallucinations. The mechanisms linking hallucinations and GMV reduction are yet to be discovered^77–79^. Congruently, the expression of auditory hallucinations alone is also related to more severe impairments already present in the retina^78^. Together, studies focused on the interconnection between visual pathological phenomena (such as visual hallucinations and illusions) and their relationship to abnormalities in other perceptual modalities may, therefore, help to reveal the basic mechanism related to schizophrenia development in general.

### Morphological and pathophysiological changes in the retina

**Fig. 1:**
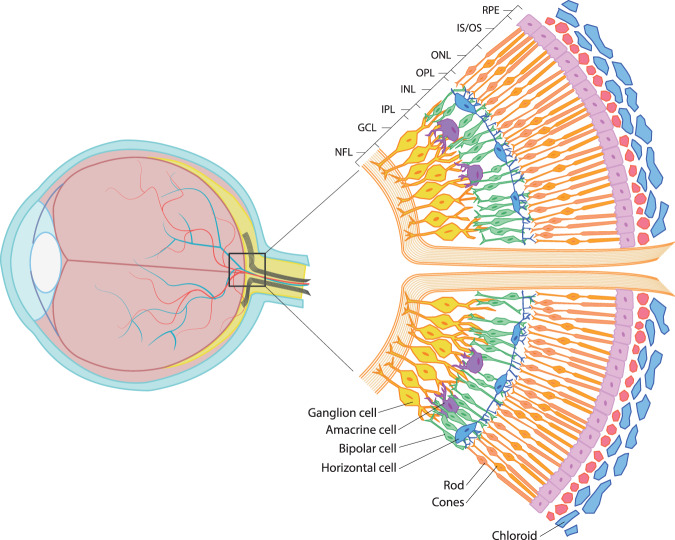
Retinal layers. The composition of the individual layers of the retina in the area of the optic nerve. NFL nerve fiber layer, GCL ganglion cell layer, IPL inner plexiform layer, INL inner nuclear layer, OPL outer plexiform layer, ONL outer nuclear layer, ELM external limiting membrane, IS/OS rod and cone inner and outer segments, RPS retinal pigment epithelium. Redrawn from retinareference.com.

In vivo studies using ocular coherence tomography (OCT) have confirmed changes in the retinal structure. The majority of studies have focused on the atrophy of retinal nerve fibers (RNFL) representing a decline in ganglion cell axons and the overall thinning of the macula^80^. Thinning of the inner plexiform layer and the inner nuclear layer (Fig. 1) in schizophrenia has also been reported^81^. Interestingly, thinning of the retina in the foveal, nasal, parafoveal, and temporal-parafoveal regions of the macula as well as a reduction in the outer nuclear and inner plexiform layers (Fig. 1) have been related to negative symptom severity (a negative score on the Positive and Negative Syndrome Scale negative subscale) and selective deficit to LSF contrast sensitivity^70^. In addition, the loss of ganglion cells in the temporal parafoveal region of the retina was associated with magnocellular ganglion cell loss throughout the disease progression^70^.

The currently open question is whether structural and functional alterations to the retina occur due to trans-synaptic retrograde degeneration originating from the regional pathology at the higher steps of visual pathway or vice versa.

Recent studies have shown that in the early stages of schizophrenia, there was a loss of GMV in the thalamus^56^. As the schizophrenia progresses, GMV loss expanded to the frontal lobes and then to the temporal lobes, occipital cortex, and cerebellum^58,82^. These studies thus suggest a retrograde nature to the retinal ganglion cell (RGC) volume loss process. When the thalamic volume decreases, it causes a loss of connectivity for RGC axons and thus their subsequent inflammation. OCT studies have shown that atrophy of ganglion cell axons and thinning of the macula manifest mostly during the chronic and long-term chronic phases of schizophrenia^83,84^, which would also indicate that a retrograde origin for retinal cell degeneration is more likely than an anterograde origin is.

Without further studies, however, we cannot rule out an anterograde nature to the process, which can be started by dysregulation of DA^66,80^ and glutamate transmission^85^. In pathological cases, both of these transmitters are capable of causing retinal atrophy and a loss of axons in specific retinal layers. This loss is thought to be caused by over-stimulation of the N-methyl-D-aspartate receptor (NMDAr). This results in an increase in the intracellular Ca^2+^ concentration in RGCs, resulting in excitotoxic damage as seen in other compartments of the central nervous system (CNS)^86,87^. Congruently, activation of GABA interneurons by nitric oxide has been proposed as a preventive mechanism of excitotoxic degeneration and a mutation of nitric oxide synthase was identified as one of the genetic risk factors of schizophrenia^88^.

Thinning of the retinal layers may also be related to abnormalities in blood supply. Recent studies using OCT angiography have demonstrated changes in retinal microvasculature in terms of both reduced perfusion and vessel density. These abnormalities are mainly associated with RNFL thinning (see above)^89^. Previous studies also observed changes in retinal venules, which dilate mainly due to chronic retinal hypoxia^90–92^. However, a similar effect on small vein widening was also observed as a result of an increased concentration of retinal DA^93^.

If we were able to understand the retrograde or anterograde origin of the onset of morphological changes, it would be possible to target therapy specifically to these sites, thereby slowing or stopping the degradation of individual cell populations of the retina, LGN, and optic nerve.

### Changes in the electrophysiology of retinal cells

**Fig. 2:**
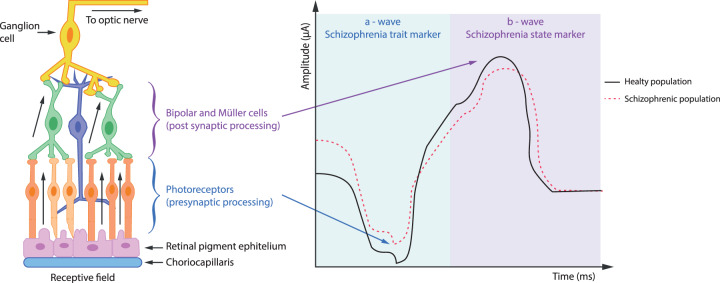
Schema of ERG signal from retinal cells. An illustration of the retina (left) and a representative ERG comparing HCs and schizophrenia patients (right). In the dark-adapted retina, a light stimulus elicits a presynaptic response from photoreceptor cells, represented by the downward-deflecting a-wave. The subsequent postsynaptic response, mediated largely by bipolar and Müller cells, produces the b-wave. The a-wave amplitude (measured from the baseline to the trough of the a-wave) depends on the intensity of the light stimulus and the integrity of the photoreceptors. The b-wave amplitude (measured from the trough of the a-wave to the peak of the b-wave) depends on the a-wave and the integrity of signal transmission within the retina. Redrawn from Hanjin Deivasse web illustration.

Morphological and biochemical changes in the retina of schizophrenia patients are accompanied by alteration in the electrophysiological response of individual retinal cells to light stimulation. Abnormalities in sensitivity to certain wavelengths, frequencies, and intensities of light during stimulation were recorded by electroretinography (ERG)^65^. These abnormalities manifest as changes in amplitude and a delayed onset of the electrophysiological response to a light stimulus (latency), but also in the structure of the a- and b-waves (Fig. 2)^94^. The largest ERG study of psychiatric disorders performed to-date (150 schizophrenia patients, 150 patients with bipolar disorder, and 200 healthy control subjects [HCs]) showed a reduction in amplitude of the a-wave and a later onset of the b-wave during stimulation focused on the electrophysiological response of cone cells in both patient groups^95^. In contrast, a reduction in b-wave amplitude, which was produced by a simultaneous response by Müller glia, responsible for the capture of neurotransmitters from intercellular space and the regulation of potassium concentration, and bipolar cells, which provide the connection between the inner and outer plexiform layer in the retina, was observed only in the schizophrenia patients. A decrease in the amplitudes of the a- and b-waves during stimulation aimed at the combined electrophysiological response of both rods and cones was also found in both patient groups. The study authors presumed that aberrations in the b-wave latency and amplitude may be considered an early and very specific biomarker of schizophrenia. Conversely, a decrease in the a-wave amplitude may plausibly be connected only to the acute phase of the disorder, as after an eight-week treatment no significant differences were observed between the schizophrenia patients and HCs^96^.

### Biochemistry of the retina

Changes in the pathophysiology of the retina in the schizophrenia population are accompanied by biochemical changes, which stem from an imbalance in excitatory and inhibitory neurotransmission, as outlined above. One of the most important catecholaminergic neuromodulators reaching the highest local concentration within the primary visual system in the retina is DA^73,86^. DA is one of the main neuromodulators in the mammalian brain and is considered, within the scope of the theoretical model of schizophrenia, one of the principal mediators of positive symptoms via the dopaminergic mesolimbic pathway, as well as negative symptoms via the mesocortical pathway^97^. Moreover, dopaminergic substances such as cocaine and amphetamine induce or trigger psychosis^86^. The concentration of retinal DA is not constant and is affected by various factors such as circadian rhythms and age^98^. Animal studies have confirmed that the concentration of retinal DA is regulated via stimulation of the hypothalamus, followed by activation of retinopetal neurons, which release histamine. The axons of these neurons run through the optic nerve to the boundary of the inner plexiform and nuclear layers. Here, the release rate of intercellular histamine, which binds itself to the D_1_R receptors of DA-releasing amacrine cells, may be increased^99,100^. However, there is only indirect evidence of this mechanism in humans. Recent methods only enable modulation, the ERG curve of the b-wave via positive stimulation through food (hypothalamus activation) or by administering the DA agonist methylphenidate^101^.

**Fig. 3:**
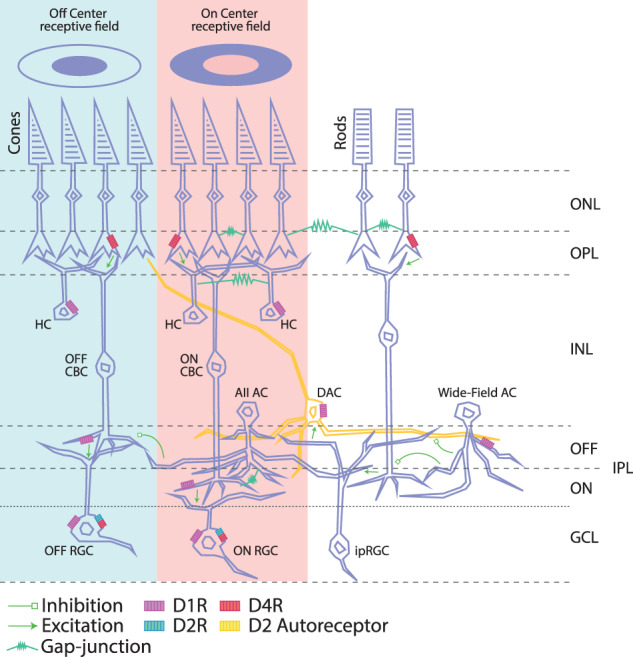
Distribution of DA receptors in retinal cells. Schematic of the retinal circuitry with cell types expressing specific DA receptors in the retina. The DA receptors D_1_R, D_2_R, and D_4_R and D_2_ autoreceptors localized on various cell types are indicated in purple, green, yellow, and red. The dopaminergic amacrine cells (DACs; orange) stratify primarily in the outermost layers of the IPL and send axon-like dendritic projections to cone terminals in the OPL and to the inner layers of the IPL, where they contact AII amacrine cells (ACs). Synaptic excitation and inhibition are illustrated by arrows and bar-line (green). Gap-junctions are shown as sawtooth symbols. The two concentric circles at the top represent OFF-center and ON-center RGCs, which respond oppositely to light in the center and surroundings of their receptive fields. DAC: aopaminergic amacrine cell, HC horizontal cell, RBC rod bipolar cell, CBC cone bipolar cell, AC amacrine cell, RGC retinal ganglion cell, ipRGC intrinsically photosensitive RGC. Redrawn from Roy & Fieldl^50^.

The majority of dopaminergic cells are located between the inner nuclear and plexiform layers of the retina^102^. They respond to DA through the metabotropic G_s_ D_1_ receptors located at the membrane of bipolar, horizontal, amacrine, and ganglion cells^103^ and via metabotropic G_i_ D_2_ receptors at the membranes of both rods and cones (Fig. 3). D_2_ receptors are also present at DA-releasing AII amacrine cells. D_2_ on these cells function as autoreceptors regulating the release of DA^104–106^.

Extracellular DA modulates the degree of excitability of retinal cells directly and indirectly. The direct connection functions via synaptic or volume transmission and bonding to D_1_ and D_2_ receptors. DA indirectly affects retinal cells in multiple ways: (1) It alters the probability of opening/closing membrane ion channels, but also the length and frequency of the opening/closing^107^. (2) It regulates the excitability of the horizontal intercellular gap junction in the inner plexiform layer, where a decrease in the DA concentration increases the permeability of the gap junction in AII amacrine cells. Their activity modulates the excitability of metabotropic ON-center bipolar cells (BCs)^108^ (Fig. 3). Excited AII amacrine cells have an inhibitive effect on ionotropic OFF-center BCs (Fig. 3) and, therefore, suppress distortion and noise at the frequencies and intensities of action potentials, which are transferred to ganglion cells^109^. Conversely, increased DA concentrations inhibit horizontal communication and the permeability of gap junctions in AII amacrine cells, while also altering the continuity of action potential changes in ON-center BCs^106,110,111^. This increases their sensitivity to local stimulation of a particular group of photoreceptors and inhibits peripheral stimulation. Fluctuations in extracellular DA concentrations also remodulate the nature of the signal exiting the RGCs and BCs and, therefore, affect the signal from ON- and OFF-centers of receptive fields^112^. Long-term pathological changes in DA concentrations may lead to a loss of spatial vision and temporal sensitivity^73^. (3) DA modulates the response of retinal GABAc receptors, which participate in the communication between retinal cones and bipolar and horizontal cells—in other words, they modulate the intensity of the signal output from photoreceptive cells via the degree of membrane hyperpolarization, which regulates its excitability^113^.

Glutamate is the main excitatory neurotransmitter in the retina and the only output neurotransmitter of all photoreceptors^114^. Glutamate is released by photoreceptors during depolarization (the phase when photoreceptors are not stimulated by light) and an increase in its concentration affects ionotropic OFF-center BCs. Conversely, stimulation of photoreceptors by light is followed by hyperpolarization, the concentration of glutamate decreases, and metabotropic ON-center BCs are stimulated. The glutamatergic system is generally related to positive, negative^70^, and cognitive symptoms of schizophrenia^83^ via hypofunction of NMDAr. Dysregulation of NMDA also leads to an increased release of DA^115^, which affects the extent of positive symptoms such as visual distortions, hallucinations, and altered performance in psychophysiological testing of visual perception^116,117^. Changes in visual perception are also connected to excitotoxic damage to photoreceptive cells and disrupt the perception of motion and high spatial frequencies of image stimuli^70,87^. Animal testing on rodents has shown that it is possible to reduce the ERG b-wave amplitude of Müller cells during artificially induced reduction of glutamate transmission via the glutamate aspartate transporter^118^.

The visual perception processes described above may be studied by administering agonists or antagonists of specific receptors. However, it is difficult to say if an observed effect occurs on the retina or downstream in the cascade of visual perception processes. Administering D_2_ antagonists (haloperidol, benzhexol, and fluspirilene) to schizophrenia patients for three weeks caused a decrease in sensitivity to contrast of visual stimulus compared with HCs. However, sensitivity was not decreased globally and depended on the stimulus orientation on the vertical or horizontal plane of the visual field^73,119^. Antipsychotics (trifluoperazine, fluphenazine, and haloperidol) also inhibited sensitivity to high and medium spatial frequencies of image stimuli. Conversely, the opposite effect was observed for LSFs^68^. In both cases, the physical saliency was affected. A general increase in sensitivity to contrast after dopaminergic stimulation via L-dopa was observed in both schizophrenia patients^120^ and HCs^121^. A hyperdopaminergic state during early phases of schizophrenia is responsible for increased sensitivity to LSFs and related excessive excitation of ganglion cells, which constitute magnocellular pathways responsible for conducting a signal to the visual cortex^122^. It is plausible that all of the aforementioned effects are associated with the ability of DA to affect the size and sensitivity of retinal receptive fields for distinct spatial frequencies^123^ via inhibition of the gap junction of retinal horizontal cells and reduction of the amplitude induced by light incident on the photoreceptors. Both of these effects would then have a physiological basis in dopaminergic mechanisms related to brightness adaptation^73^.

It is important to emphasize that the retina is considered a part of the CNS, as during embryonic development it originates from the same tissue as the brain and shares with it many biological processes, including the role of neurotransmitters and their receptors, lateral connectivity, and feedback mechanisms^124^. Therefore, changes in retinal function may be caused by schizophrenia itself, its course, and antipsychotic medication^124,125^. Some of the observed retinal dysfunctions may be related to other factors and comorbidities, such as systemic diseases (diabetes, hypertension), smoking, antipsychotic medication, drug abuse, sex, obesity, attention span, degree of arousal, and motivation, as they influence the retina via histaminergic and serotonergic inputs from brain regions^49^.

The pathology of retinal function may be generally characterized in two basic categories: (1) hypofunction caused by damaged retinal cells due to glutamate dysregulation, and (2) hyperfunction due to excessively high DA concentration. However, both of these effects cause a modulation of optosensoric signals, which are transferred further to higher levels of precortical and cortical visual processing circuits.

### Optic nerve and LGN

**Fig. 4:**
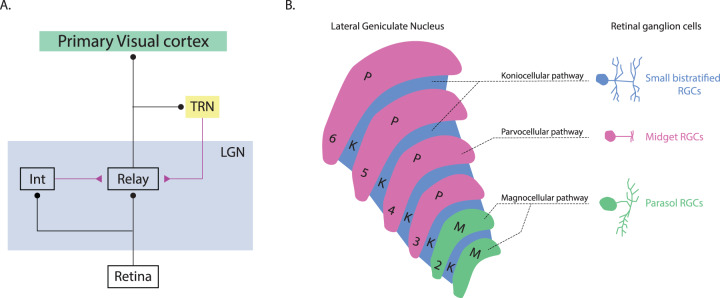
Structure of the LGN with three distinct layers. Simplified diagram of visual thalamic circuitry and the LGN. **A** Diagram of feedforward and feedback inhibitory pathways that influence LGN RCs. Excitatory inputs are indicated in purple. Inhibitory inputs are indicated in black. TRN: thalamic reticular nucleus; LGN lateral geniculate nucleus; Int LGN interneuron. Modified from Casagrande & Xu^129^. **B** LGN diagram with ganglion cells type. RGCs retinal ganglion cells. Redrawn from Kim et al.^141^.

A small proportion of the optic fibers are diverted from the retina into the retinohypothalamic tract, which leads to the anterior hypothalamic nucleus. This connection affects pupilar dilation (sympathicus) and constriction (parasympathicus)^126^. However, up to 90% of the signal is passed through ganglion cell axons, forming three independent pathways (magno-, parvo-, and koniocellular) inside the optic nerve, into the optic chiasm, where some of the nerve fibers cross, and further into the LGN of the thalamus^127^. According to recent findings, the regulation, timing/distribution, and strength of signal input from the retina into specific parts of the primary visual cortex (V1) occur within the LGN^128^. However, regulation of the output signal from the LGN is a very complex process regulated by several feedforward control mechanisms. The most prominent non-retinal inputs, which also react to the output signal from the LGN, are glutamatergic inputs from the cell of the VI layer of the V1 operating on both the ionotropic and metabotropic glutamate receptor pathways and directly affecting the depolarization of relay cell (RC) membranes in the LGN. In principle, the LGN consists of two cell classes. First, the glutamatergic RCs, which send axons to the visual cortex, and second the interneurons (INs), the axons of which remain in the LGN (Fig. 4a). The visual signal in the LGN is regulated by local inhibitory neurons and thalamic reticular nucleus (TRN) inhibitory neurons. These neurons are connected by feedforward and feedback circuits (Fig. 4a). The feedforward pathway consists of the classic triad synapse^129^. The afferent axons of the optic nerve connect to the dendrites of LGN INs and RCs. The INs and RCs then form a dendro-dendritic connection at the same synapse. In the case of feedback, the TRN inhibitory neuron receives a signal from the axon of the RC. The TRN neuron sends an inhibitory connection back to terminate on the dendrite of the same RC (Fig. 4). Both of these inhibitory circuits also contribute to the character of the signal that is going through the RCs^128,130^.

Further modulation of the output signal comes from cholinergic endings innervating the INs and RCs of the LGN^131^. The contribution of cholinergic transmission to the overall nature of the signal emanating from the LGN is considerably complicated. This is because of the presence of slow metabotropic (M1) and fast ionotropic muscarinic ACh receptors in the membranes of RCs (in both cases, their activation leads to depolarization)^132^. Additional ACh M2 receptors are present in INs and TRN cells and their activity leads to hyperpolarization. Overall, however, we can say that ACh inputs to the LGN have an excitatory effect. RCs are depolarized and inhibitory feedforward and feedback circuits are blocked^128^.

In the case of DA, the density of dopaminergic innervation is lower in the LGN compared to the rest of the thalamus^133^. Animal studies have demonstrated the presence of D_1_ and D_2_ receptors in the membranes of LGN RCs. Stimulation of D_1_ receptors led to inhibition of their excitability. Conversely, activation of D_2_ receptors had an excitant effect on their glutamatergic synapses^134^ and, therefore, the overall sensitivity to local contrasts within the framework of visual perception was increased^128,135–137^.

Like the retina and other parts of the visual system, the LGN shows the presence of receptive fields responding to specific aspects of the visual scene^138^. Recent studies have shown possible modulations of receptive fields based on feedback from the V1^139^. However, a key outstanding question, particularly for understanding visual impairment in schizophrenia, concerns the influence of monoamines and ACh on this modulation.

### Magnocellular, parvocellular, and koniocellular pathways

The morphological structure of the LGN is characteristically constituted of visible distinctive layers, which reflect the structure of the optic pathway. These layers are composed of three separate nervous pathways, which are divided into twelve layers (four dorsal parvocellular [PC], two ventral magnocellular [MC], and six koniocellular [KC] interlayers; Fig. 4). The individual retinal input pathways differ not only in the sensitivity of their cells to the spatial frequencies of an image, electromagnetic spectrum wavelengths, and contrast, but also their own physical morphology^140^.

The majority of the MC pathway consists of axons of parasol RGCs^141^. The primate retina is composed of two types of parasol puncta: ON-parasol cells depolarizing when light strikes the center of their receptive field and OFF-parasol cells with the opposite reaction^142^. These cells have a larger dendritic field (30–300 μm) compared to midget RGCs and their input signal is ca. 80% composed of amacrine cell activity with BCs contributing the remainder of the signal. MC pathways react to the velocity and direction of a moving object—its spatial localization. They are sensitive to low contrasts and LSFs. On the other hand, they have high temporal resolution. The proportion of the input signal from rods and cones to the MC pathways depends largely on the light conditions^143^ (Table 1). They assist in stereopsis, depth perception, hyperacuity, and recognition of objects in a visual scene, including associations between them and separating individual objects from the background^141,144^. They play a central role in the perception of the overall organization of the stimulus^145^. MC pathways are highly myelinized and signal transmission to the visual cortex is considerably faster compared to the two other pathways. These pathways also play a key role in directing eye movements and in the coordination between our body and moving objects^139^.

**Table 1:** Morphological and functional characteristics of the visual pathway.

Characteristic	Magnocellular	Parvocellular	Koniocellular
Ultimate destination in the brain	Predominantly parietal lobe	Predominantly temporal lobe	Probably the V1
Sensitivity to movement and flicker	Very sensitive	Insensitive	Not sufficiently described
Spatial frequency summation	Non-linear	Linear	Linear
Ability to resolve details	Good at resolving coarse detail	Good at resolving fine detail	Overlap of M and P cells
Ability to detect contrast	Sensitive to low contrast objects	Sensitive to high contrast objects	Overlap of M and P cells
Effect of blur	Relatively insensitive to blur	Greatly affected by blur	Not sufficiently described
Area of visual field where most sensitive	Peripheral vision/ large	Central vision/ small	Not sufficiently described
Ability to discriminate colors	Color insensitive	Color sensitive	Yellow and violet-blue sensitive
Spatial frequency	Low	High	Overlap of M and P cells
Temporal frequency	High	Low	Overlap of M and P cells
Response latency	Short	Long	Medium
Temporal resolution	Fast	Slow	Medium
Dendritic field size (μm)	30–300 μm	10–100 μm	Not sufficiently described

The signal passing through MC pathways is further processed and continues into specific areas of the V1. However, some recent studies have questioned the continuation of magnocellular pathways into the dorsal stream, based on the coactivation of ventral stream regions by low spatial frequencies in some specific visual tasks^146,147^.

**Fig. 5:**
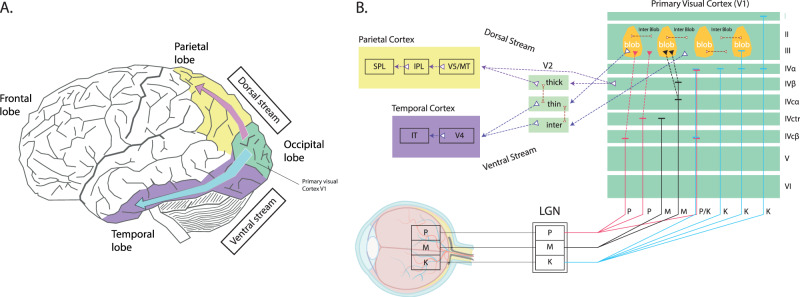
Visual pathways and brain streams. **A** The ventral (purple) and dorsal (yellow) streams of visual information processing. **B** A detailed scheme of signal distribution from PC, MC, and KC visual pathways to the LGN and further to the primary (V1) and secondary (V2) visual cortex and subsequently to the dorsal or ventral stream. Redrawn from Casagrande & Xu^129^.

PC pathways are predominantly composed of midget RGCs. These cells have small bodies and their dendritic branching is only about 5–10 μm in diameter in the central part of the retina (it can reach up to 225 μm in the peripheral regions)^141,148^. This corresponds to smaller receptive fields. Midget cells are mainly localized in the central part of the retina and form a one-to-one connectivity with the midget BCs that receive the signal from the single cone^149^. As with parasol RGCs, midget RGCs have ON- and OFF-center types. PC pathways are sensitive to colors, high spatial frequencies, shape, and other details of objects in a visual scene (Table 1). Their speed of transferring nerve impulses and degree of myelinization are lower. The summation of their membrane potentials is linear with a low action potential velocity (Table 1). They are also able to react during the entire effect duration of a stimulus. PC pathways end mainly in the lower parts of the IVC layer V1 (IVCβ and IVCctr). A smaller proportion of their endings are also located in the IVA layer (Fig. 5).

KC pathways are predominantly composed of axons of small bistratified RGCs^150^. These cells are assumed to have a supportive function for color vision with low spatial resolution^141^. Animal studies performed on primates showed the reaction of some KC cell groups within the LGN to chromatic stimuli, violet-blue (400–470 nm) and yellow (600 nm) wavelengths, and brightness^151–153^. KC pathways end in cytochrome oxidase blobs contained in the I, III, and IVα V1 layers. Some parts of these blobs are also present in the structure of the VI layer of the V1. However, the exact function of the KC interlayers in humans is not yet fully understood^154^.

It is useful to recall that the size of the receptive field plays an important role in sensitivity to specific frequencies of image perception^155,156^. This sensitivity is to some extent determined by the specific morphology of each class of RGCs, specifically by the size of their dendritic field. In normal physiological conditions, receptive fields are fine-tuned by DA, which allows adaptation to the specific light conditions of the surrounding world^106,157^. Future research should answer the question of how much DA and retinal morphological changes in schizophrenia patients alters the sensitivity of the human receptive field and how these changes affect the spatial integration of visual perception in higher precortical and cortical areas.

## Cortical integration and processing of visual stimuli

Earlier studies pointed to the central role of MC pathways in the disruption of visual perception in the schizophrenia population, predominantly based on reduced contrast sensitivity at LSFs^6,8,13,45^. This approach was later criticized for several reasons^32^, in particular, the uncertainty that the stimuli used in the studies really activated only the MC pathways. Another point was the lack of distinction between the subcortical MC pathway and the cortical dorsal stream (Fig. 5)^26,146^. Thus, the overall disruption of visual stimulus integration in the cortical areas is currently attributed to gain control mechanisms^112^. The latter at the molecular level is related to the ability of signal integration on pyramidal neurons and its modulation within the feedback and feedforward circuit. The core modulator in this case is thought to be DA^31^. DA at the cell body increased the influence of bottom-up inputs through a combination of augmenting a slow, depolarizing influence (Na+) and decreasing a slow, hyperpolarizing current (K+)^31,158^.

In general, visual processing consists of a set of mechanisms optimizing perception of visual information further utilized in goal-directed behavior. The quality of the perceived information is controlled from both directions, ascendentally (bottom-up) and descendentally (top-down). Throughout visual processing, the gain of information is controlled precisely and the information from the lower levels of the visual system is integrated on every level of processing^6^. Precortical processing is performed during the course of projection from the retina to the V1 and in subcortical circuitries participating in higher cortical processing. Subcortical structures cooperating on higher processing include higher order thalamic nuclei (pulvinar, mediodorsal), the basal ganglia, and the amygdala. Higher order thalamic nuclei participate in the integration of cortical information and the reconnection of distinct cortical compartments or working memory^159,160^; the basal ganglia contribute to filtration of information, working memory, and attention^161^; and the amygdala cooperates on information contextual analysis or shifting visual attention towards emotional stimuli^162^. In addition, the thalamic function is strongly influenced by monoamines and ACh^128^. These neuromodulators amplify the bottom-up and top-down signal-to-noise ratio^163^.

Top-down control is assumed to be initiated by approximate information about an object carried rapidly via MC pathways to the visual cortex and through the dorsal stream to the PFC. Complementary to that, the PC pathway carries more detailed information about visual stimuli in a slower manner to complete and specify the image^164,165^. The two pathways cooperate and coordinate with each other^166^. The theory of a direct pathway from early visual areas to the PFC corresponds to immediate reactivity of PFC areas to visual stimuli. Together with early visual areas, the caudal middle frontal cortex was activated. This cortex includes the frontal eye field (FEF), orbitofrontal cortex (OFC), and ventromedial PFC^167^. Some studies have proposed a direct connection of the MC pathway to the lateral PFC^164^. As the brain structure responsible for executive functions, planning, and making decisions, the PFC analyzes the inner and outer contexts of information and provides top-down control over other brain areas and neural networks and their synchronization^168^. The FEF controls saccades and was shown to have a direct projection to the V4^169,170^. The OFC and ventromedial PFC connect with the amygdala and process emotional stimuli. The FEF, OFC, lateral PFC, and ventromedial PFC all have connections to the inferior temporal cortex (IT), a key area for integration, semantic memory, and recognition^171^. The PFC is assumed to project the information received from the MC pathway to the IT, which afterwards categorizes the approximate information and projects the integrated information back to the occipital cortex to sharpen the attention and acquire the most relevant information about the object of observation. fMRI studies focusing on the perception of visual illusions have shown impaired top-down processing (in the frontoparietal network) in patients with schizophrenia and a predominant emphasis on the integration of bottom-up sensory stimuli^26,38,172^.

The process of integrating visual information consists of excitatory and inhibitory projections, when the purpose is usually to enhance perception about the object of interest while simultaneously suppressing perception of the surroundings. Nonetheless, the surroundings can have a major impact on the accuracy of object identification^173^. Higher processing of visual information includes executive functions, in which we see impairment in schizophrenia patients, such as working memory^174^, long-term memory and learning^175^, object^176^ or facial recognition^177^, and context integration^26^.

Abnormalities in higher cognitive processing lead to the creation of an abnormal perception of surrounding reality, which in turn supports abnormal perception in a positive feedback loop. Imbalance in bottom-up and top-down visual processing affects selective attention, visual working memory, object and facial recognition, and memorization of visual information^164,178,179^. In schizophrenia, imbalances in bottom-up and top-down processing create conditions for symptoms consisting of visual distortions, alternated perceptions of illusions, visual hallucinations, and cognitive impairments including social cognition^180,181^. It is possible to start considering the connection between perceptual disorders and cognitive dysfunctions or specifically to consider cognitive dysfunction as a consequence of long-term imbalances in the signal-to-noise ratio of sensory modalities of visual perception.

### Instability of the inner world model as a schizophrenia trigger

Visual perception, the dominant source of information in the development of our inner world model, modulates our experience of reality. The disruption of visual perception modalities in schizophrenia may contribute to the development of an incorrect model of reality^182^, which further accelerates the development of the disease itself.

As mentioned above, schizophrenia is characterized by instability in the input visual signal, with hypersensitivity to LSF in the early stages of the disease (before and during the first episode). It is followed by a progression to hyposensitivity and affects other frequencies in the visual field. The instability of the input signal (bottom-up) then leads to biased prediction models; more precisely, the unstable signal-to-noise ratio does not allow the creation of a stable/dominant model that would adjust intrinsic reality predictions and contextual modulation.

**Fig. 6:**
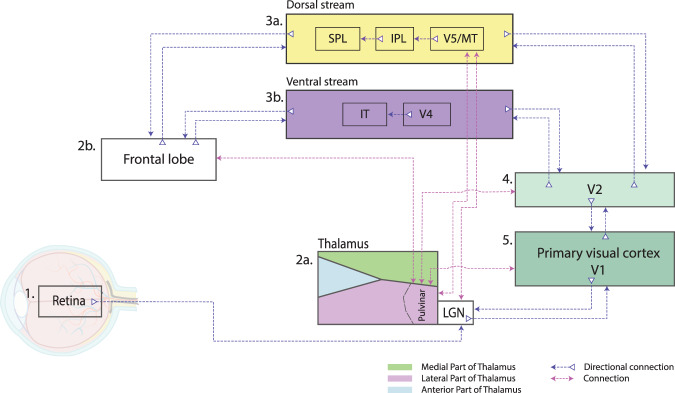
Aberrant signal propagation and subsequent physiological changes. This simplified scheme illustrates the hypothesis of the early spread of an aberrant signal within the visual circuit and the physiological changes associated with it. **1** In the early stages of the disease, an aberrant signal is formed on the retina and further propagates within the precortical and cortical visual circuit. **2a/b** The first areas that are likely to fail to adapt to the unstable signal and where there is GMV thinning are the thalamus and areas of the frontal lobe. **3a/b** From there, the pathophysiological changes spread into the lower visual processing areas (**4** and **5**). SPL superior parietal lobule, IPL inferior parietal lobule, V5/MT middle temporal visual area, IT inferior temporal cortex, V4 visual area 4, V2 secondary visual cortex, LGN lateral geniculate nucleus.

The most probably scenario is as follows: The long unstable and noisy signal from the visual periphery is transmitted to other areas within the precortical circuit. These areas modify the primary noisy signal and abstract the outputs for higher cortical areas. In cortical pyramidal neurons (PNs), further contextual modulation/abstraction of the signal occurs^31^ in terms of suppression, amplification, or synchronization^26^. Higher cortical areas, led by the PFC^183^, make predictions based on this signal^183^. However, the formation of long-term stable predictions is suppressed by variable and unstable noise from lower areas of the perceptual cascade. We speculate that the demanding process of adaptation to this noise signal in higher cortical areas may in the long run lead to a neurotoxic process connected to reduced connectivity among PNs, preventing the formation of stable representations. Gray matter reduction, which is strongly associated with schizophrenia, is attributed to an overall reduction in the number of synapses on the PNs^184^. These changes may then propagate back to lower stages in the perceptual cascade, adding new noise to the already noisy signal. This time, however, due to the reduced synaptic connectivity (Fig. 6).

The loss of neural connections in the PFC is also influenced by genetic factors such as gene expression in inhibitory GABAergic INs. Suppression of GABAmergic INs leads to a decrease in gamma synchrony affecting synapse formation and stability^184,185^. Thus, in schizophrenia patients, reduced connectivity in the cortex could be compounded by the addition of an unstable signal from the periphery that forces a new network modulation based on its instability.

Based on these considerations, we hypothesize that increased noise in the signal from the sensory periphery may serve as a trigger mechanism for the development of schizophrenia. This assumption is indirectly supported by the probable protective effect of congenital blindness or early cortical blindness in the high-risk population^186,187^, even after taking the low incidence of these health conditions in the general population into consideration^46,186–188^. There are no known schizophrenia cases in people who have suffered congenital blindness or lost vision at a very early age. Traditionally, there have been three main hypotheses^46,189^ attempting to explain this phenomenon: (1) Blindness eliminates abnormal visual percepts, which are able to disrupt visual perception and, therefore, mental models of the world created on its basis. (2) Visual impairment can improve some aspects of sensomotoric, olfactory, and auditory cognition—the modalities of perception that are disrupted by schizophrenia—and this causes a compensation effect. This effect can protect against schizophrenia only if the vision loss occurs within the first year of life. (3) Congenital blindness is also connected to a reduction in language flexibility and dynamic representation of the body, which probably provides a protective effect regarding the experience of the self. We propose a new (fourth) hypothesis that the blindness-mediated suppression of aberrant visual signals from the sensory periphery prevents the amplification of network instability in higher cortical areas.

## Conclusion

The disruption of the early stages of visual processing and related mechanisms of higher visual cognition in schizophrenia patients has been described repeatedly. The incorrect integration of visual information occurs even in the early phase of visual perception. Visual information is subsequently coded into a specific pattern of neuronal signal. The disruption is detectable in both the retina and other segments of the visual cascade, such as the optic nerve, the LGN, and the V1.

In the early stages of the disease, and in untreated patients, hypersensitivity to LSFs has been documented. During the further course (and medication) of schizophrenia, this hypersensitivity turns into hyposensitivity and begins to affect other spatial frequencies of visual perception. Alterations to the visual signal, which are largely inconsistent over the course of schizophrenia (remission and relapse phases), may lead to the formation of inconsistent internal models of the world. These signal alterations (noise-to-signal ratios) are associated with fluctuations in DA and ACh levels, decreased activity of inhibitory GABAergic INs, and hypofunction of NMDAr associated with gradual loss of cell populations in the precortical visual circuit. The volatile and noisy signal from the periphery may then act as an amplifier of primarily decreased connectivity within frontal areas, which may then prograde retrogradely to lower cortical areas of the visual information processing circuit.

This assumption opens several important questions to be addressed in future studies. First, the role of disruptions in visual signal integration in the interactions between different regions of the precortical and cortical circuit should be elucidated. Second, the influence of error generation in regions of upstream visual pathways on the overall interaction among them should clarify the aberrant processing of visual information. Importantly, these errors could be cumulative, compensatory, or both. Third, the association between unstable signal from the visual periphery and gray matter loss in cortical areas should be verified. Answering these questions could identify novel possibilities for the treatment and remediation of schizophrenia. For example, specific modulation of the visual scene (noise, contrast, etc.) could be used to improve its integration within visual processing in schizophrenia patients or high-risk subjects by compensating for the initial steps of the pathophysiological cascade.
